# Harmonizing biosynthesis with post-ingestive modifications to understand the ecological functions of plant natural products

**DOI:** 10.1039/d2np00019a

**Published:** 2022-05-16

**Authors:** Jiancai Li, Ian T. Baldwin, Dapeng Li

**Affiliations:** CAS Key Laboratory of Insect Developmental and Evolutionary Biology, CAS Center for Excellence in Molecular Plant Sciences, Shanghai Institute of Plant Physiology and Ecology, Chinese Academy of Sciences Shanghai China jcli@cemps.ac.cn; Max Planck Institute for Chemical Ecology, Department of Molecular Ecology 07745 Jena Germany baldwin@ice.mpg.de; National Key Laboratory of Plant Molecular Genetics, CAS Center for Excellence in Molecular Plant Sciences, Shanghai Institute of Plant Physiology and Ecology, Chinese Academy of Sciences, CAS-JIC Center of Excellence for Plant and Microbial Sciences (CEPAMS) Shanghai China dpli@cemps.ac.cn

## Abstract

Covering: up to 2022

The recent dramatic advances in our understanding of the biosynthetic pathways that produce diverse bouquets of plant-derived natural products have far surpassed our understanding of the function of these compounds for plants: how they influence a plant's Darwinian fitness in nature. Our understanding of their mechanisms, the life-processes targeted by these compounds, is similarly poorly resolved. Many plant specialized metabolites (PSMs) are further modified after ingestion by herbivores, and these post-ingestive modifications are frequently essential for PSM function. Here we summarize the biosynthesis and functional mechanisms of 17-hydroxygeranyllinalool diterpene glycosides in the ecological model plant *Nicotiana attenuata*, and summarize the post-ingestive modifications known from other two-component PSMs. We propose that parallel comparisons of plant natural product biosynthetic pathways and insect post-ingestive metabolism of the same plant tissues (“frassomics”) will facilitate the often-elusive identification of the molecular targets of these effective chemical defenses, contribute to elucidations of post-ingestive metabolite interactions in insect guts, and predicate the rapid evolutions of resistance against insecticides inspired by PSMs. We highlight the value of conducting these parallel investigations at the level of the entire metabolome so as to include the multiple interacting pathways in both natural product biosynthesis as well as their post-ingestive processing. We introduce the concept of frass metabolite QTL (fmQTL) analysis that integrates powerful forward genetic approaches with frassomics, and suggest that insect-guided high-throughput forward- and reverse-genetics approaches in natural habitats will advance our understanding of PSM biosynthesis and function.

## Abstract 2

The post-ingestive modifications in herbivores are essential to the function of plant specialized metabolites. Here, we highlight recent advances and discuss the prospects of functional study to plant natural products.

## Graphical abstract

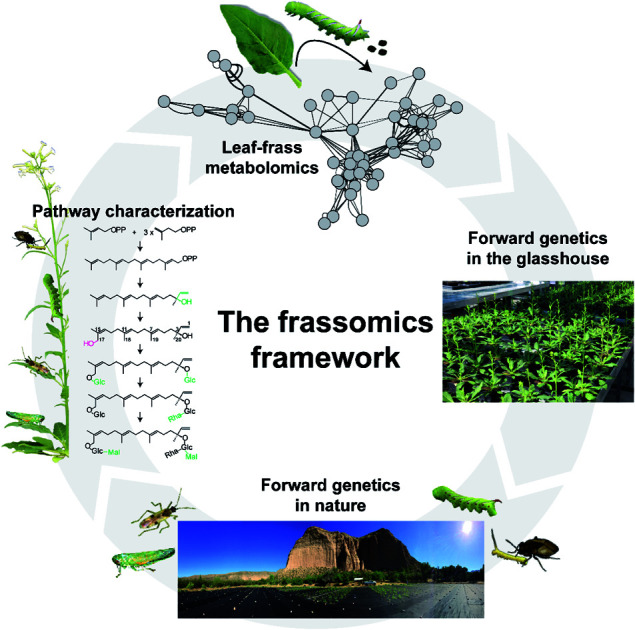

## 1 Introduction

Unlike animals that can move freely to hunt for food and escape from danger, plants are largely sessile and must cope with all the challenges that occur in habitats in which they initiate growth. Metabolites, mainly plant specialized metabolites (PSMs), play a central role in how plants find solutions to these challenges. Plants produce hundreds of thousands of PSMs, most of which are low molecular weight organic compounds (usually less than 1500 Da) that play essential roles in solutions to environmental challenges, including in defense against herbivores and pathogenic microbes, in the attraction of pollinators and carnivores, and in coping with abiotic stresses such as drought, irradiation and extreme temperatures.^1^ These metabolites have long been considered to be “secondary” metabolites, not required for growth and development, in contrast to “primary” metabolites, such as amino acids and glucose, which clearly are. Recent research has uncovered many primary functions for these metabolites, as cogently reviewed by Erb *et al.*^2^ As a consequence, the appellation “secondary” has increasingly been replaced by “specialized” for PSMs, to highlight another characteristic of these metabolites, namely their restricted taxonomic distributions, a trait which also highlights their likely diverse functional roles in specific ecological contexts.

The therapeutic properties of plants, mainly contributed by PSMs has long been recognized in traditional herbal medicine.^3^ Most modern pharmaceutics and insecticides have been inspired by the active moieties of the natural products of plants and microorganisms.^4^ Frankel's seminal *raison d'etre* publication^5^ reminded ecologists of an earlier literature that recognized the importance of PSM in coping with environmental stresses.^6^ Dealing with attack from herbivorous insects is one of these essential functions of PSMs, as suggested by co-evolutionary arms race theory,^6^ which posits that as plants evolve new defenses, insects evolve countermeasures or co-opt plant defenses for host location and defense against their natural enemies.^7,8^ For example, the pyridine alkaloid, nicotine, from *Nicotiana* plants is a neuromuscular toxin effective against all animals with cholinergic neuromuscular junctions, including herbivorous insects.^9^ However, the Lepidopteran specialist larvae of *Manduca sexta*, are unusually tolerant of nicotine ingestion and even co-opt nicotine to repel natural enemies.^10^ In response, plants perceive signals from larval oral secretion and attenuate the accumulation of nicotine through ethylene signaling,^11,12^ as well as increase the production of proteinase inhibitors which synergistically enhance this nicotine-based defense.^13^ These co-evolutionary dynamics between plants and insects are thought to accelerate the evolutionary diversification of PSMs.^14,15^ Research into the sophisticated and multiple functions of PSMs will greatly facilitate our understanding of the evolutionary origins and functional consequences of plant specialized metabolism, and how they can be more effectively applied in pest management in agriculture. However, the vast majority of these PSM functions have only been evaluated in laboratory and glasshouse experiments with limited environmental complexity, with few functions having been verified in natural habitats.^16^

Thanks to the awesome developments in mass spectrometry, functional genomics and synthetic biology, the structure and biosynthetic pathways of PSMs that are important for medicinal and agricultural applications are being rapidly elucidated.^17^ However, the PSMs that accumulate in plants are rarely metabolically inert, frequently requiring activation during an ecological interaction, for example, with herbivorous insects, with continuing reactions that occur when PSMs are ingested. Post-ingestive modifications are rarely studied, but recent research suggests that these modifications are directly associated with their ecological functions. The post-ingestive modifications can function as detoxifications, that alleviate the toxic property of PSMs, or as activations that enhance their toxic properties. The commonly deployed detoxification and metabolism strategies of insects towards ingested PSMs have been well-reviewed and can be summarized by three sequential steps: metabolism of toxic moieties (phase I); hydrophilic conjugation to increase water solubility (phase II); and excretion (phase III).^18,19^ Here we focus on the metabolism that occurs in insect guts during digestion prior to absorption into gut cells and highlight recent research with the diterpenoids that accumulate in a wild tobacco plant, *Nicotiana attenuata*, to prognosticate about a new post-ingestive-metabolism-centered methodology that will likely drive future advances in our understanding of the plant-insect interaction.

### 1.1 Natural-history-driven exploration of natural product biosynthesis and function in *Nicotiana attenuata*

The wild tobacco, *N*. *attenuata*, native of the Great Basin Desert in the southwestern United States, thrives in a hostile desert environment, in part by producing a diverse portfolio of specialized metabolites, that includes pyridine alkaloids, phenolics, phenolamides, terpenoids, acylsugars and lipid-derived volatile organic compounds. The functions of these metabolites have been studied in a research program designed to train molecularly-enabled field biologists, namely “natural history motivated gene function” program, in which “research projects start with hypotheses originating from field work and end with tests of function, carried out after the release of transformed plants at the field station”.^20^ This program is commonly realized in 4 sequential steps: (a) an observation of a plant-insect interaction in a natural habitat that suggests its mediation by PSMs; (b) elucidation of the specialized metabolite biosynthetic pathway for the particular PSM and producing several lines of transgenic plants in which key biosynthetic genes are silenced and/or over-expressed to alter PSM accumulation patterns; (c) release of the transgenic plants into the plant's natural habitat to query a diverse suite of organisms that naturally interact with the plant (herbivores from many feeding guilds, pollinators, mycorrhizae, associated bacteria, natural enemies) about PSM function; (d) deeper characterization of candidate functional mechanisms with repeated cycles of experimental tests of function in the laboratory, glasshouse and field. This “natural history motivated gene-function” research program deeply integrates molecular mechanistic studies with ecological and natural history based functional studies, and will facilitate our understanding of the chemistry that mediates plant–herbivores interactions and contribute to sustainable agricultural practices.

Pyridine alkaloids, including nicotine, and 17-hydroxygeranyllinalool diterpene glycosides (HGL-DTGs) are two of the most abundant PSMs that accumulate in *N. attenuata*, but they represent two distinct defense mechanisms against herbivores: specialized and generalized targets for PSM toxicity.^21^ Nicotine, which is uniquely accumulated in members of the *Nicotiana* genus, is a neurotoxin that poisons nicotinic acetylcholine receptors of neuromuscular junctions, a target shared by all mobile animals but well tolerated by most plants, in part, due to their lack of muscle tissues. Thus, nicotine is a specialized-target PSM. Another classical example of PSMs with specialized targets are the phytoecdysteroids that function as analogues of the insect molting hormone, 20-hydroxyecdysone; their ingestion disrupts molting and metabolic coordination of developmental process, often resulting in death.^22^ In contrast, HGL-DTGs are PSMs with more generalized metabolic targets, that are universally distributed across large lineages, including plants, and hence require particular solutions to avoid auto-toxicity. The defensive function of HGL-DTGs results from their ability to inhibit the activity of ceramide synthase (CerS) in the biosynthetic pathway of sphingolipids, which are essential components for all eukaryotic organisms.^21^ HGL-DTGs are stored in plants in an inactive form, and require activation by a process of nonspecific hydroxylation at several different positions of the aglycones that occurs in the guts of insects, although the enzymes that mediate these hydroxylations have yet to be identified.^21^ This two-component reaction appears to be a defense strategy for the generalized-target PSMs to avoid auto-toxicity, such as glucosinolates, cyanogenic glucosides, benzoxazinoids.^23^ The two components mainly include inactive toxic substrates and “detonators” that are spatially or temporally separated, with the toxin being released only in response to damage which brings the separated components together.

Most inactive forms of PSMs are glucosides, with glucosidases being the detonators. The activation of HGL-DTGs in *N. attenuata* differs from this pattern, in being mediated by a hydroxylation process, despite HGL-DTGs also being glycosides. Prior to the ingestion of *N. attenuata* leaves by an herbivore, the diterpene alcohol, geranyllinalool, is hydroxylated specifically at C-17 of the acyclic backbone by cytochrome P450 (NaCYP736As),^21^ subsequently glycosylated on the hydroxyl moieties at C-3 and C-17 by uridine diphosphate glycosyltransferases (UGT74P),^24^ and finally malonylated at the glucose/rhamnose moieties.^25^ When this particular biosynthetic sequence is disturbed, such as by the silencing of *CYP736A* or *UGT74P*, the intermediates, geranyllinalool and 17-hydroxygeranyllinalool (17-HGL), accumulate in plants, respectively. However, the accumulated geranyllinalool and 17-HGL themselves do not inhibit the activity of CerS, and 17-HGL is also not toxic to the larvae of *M. sexta*.^21,24^ The theoretical de-glycosylated product of HGL-DTGs, 17-HGL, was not detected in the frass of insects fed on HGL-DTG-producing *Nicotiana* plants.^26^ These results suggested that the defensive mechanism of HGL-DTGs differed from other two-component glycosides.

**Fig. 1:**
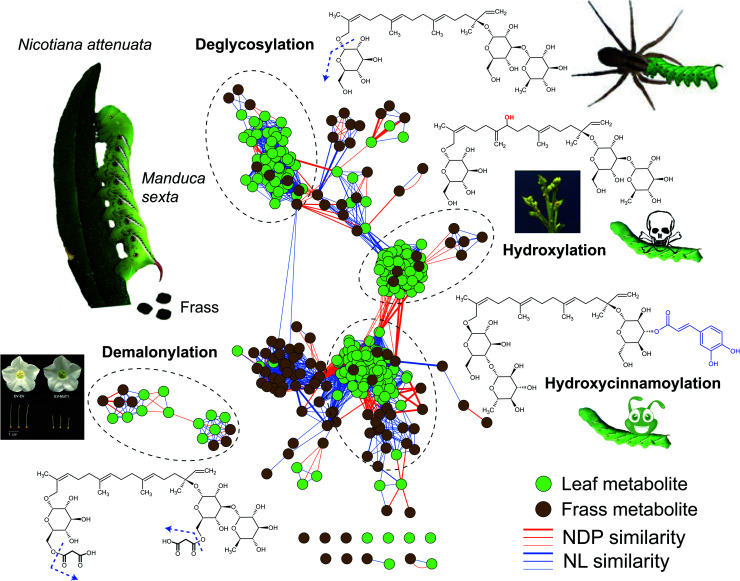
Frassomic analyses elucidate post-ingestive modifications of HGL-DTGs and their diverse functional consequences. When *Manduca sexta* larvae consume leaves of their native host plant, *Nicotiana attenuata*, they produce frass, which can be subjected metabolomics analyses and compared with similar analyses of the ingested leaves. HGL-DTGs metabolites from leaves (green) and frass (gray) are clustered into different groups based on their neutral loss (NL) and normalized dot product (NDP, representing mass fragment) similarities. Line thickness reflects the degree of similarity among different compounds. Deglycosylation of ingested HGL-DTGs reflects a detoxification process for the larvae but has the downside of increasing larval vulnerability to spider predators.^26^ Hydroxylations of ingested HGL-DTGs results in active toxins responsible for the compounds defensive function against herbivores as well as their toxicity to the producing plants, when the normal patterns of hydroxylation or glucosylations during their biosynthesis are abrogated.^21,24^ Hydroxycinnamoylations thwart the toxic effects of both ingested HGL-DTGs and chlorogenic acid,^29^ and functions as a detoxification process by mechanisms that require further study. Demalonylation occurs in the alkaline environment of the larval midgut without noticeable functional consequences for the herbivore, but when malonylation steps in the biosynthesis of HGL-DTGs in the plant are thwarted, normal stylar development is abrogated, revealing a developmental role for pathway intermediates.^25,26^

Using untargeted frass-metabolomic analysis (aka, “frassomics”), a class of novel hydroxylated HGL-DTGs were identified from the frass of *M. sexta* larvae that fed on *N. attenuata* plants.^21^ The logic of the frassomics analysis involves the calculation of structural similarities among all compound-derived MS/MS spectra by considering their common mass fragments and neutral losses, clustering of these spectra into different groups, and annotating structures of unknown compounds from their adjacent neighborhood of known compounds^27^ (Fig. 1). Subsequent structural analysis by nuclear magnetic resonance (NMR) confirmed the structures of those annotated in the frassomics analysis, that the hydroxylations were not fixed at specific carbons of the diterpene backbone (17-HGL) of HGL-DTGs, but with multiple isomers.^21^ Importantly, these hydroxylated HGL-DTGs were found to inhibit the activity of insect CerS activity and thwarted the growth of *M. sexta* larvae. These non-specific hydroxylation products of geranyllinalool and 17-HGL also accumulated in *NaCYP736A*- and *NaUGT74P*-silenced plants, and were found to be responsible for the inhibition of plant CerS activity and the clear auto-toxicity phenotype of plants interrupted in HGL-DTG biosynthesis. It is likely that the enzyme(s) responsible for the non-specific hydroxylation in the guts of *M. sexta* larvae originate from plants, as adding purified HGL-DTGs to artificial diet does not inhibit insect growth and hydroxylated products are not found in the frass of these larvae.^21^ However, the exact plant-derived detonators responsible for this toxin activation process has yet to be elucidated.

In addition to hydroxylation, the responsible mechanism of the defensive function of HGL-DTGs against *M. sexta*, de-glucosylation of HGL-DTGs also occur in the larval guts, but in a well-controlled manner. Specifically, only the glucose at the C-17, but not at C-13, of HGL-DTGs is removed by a beta-glucosidase in the midguts of *M. sexta* larvae.^26^ However, this de-glucosylation is likely a detoxification process evolved by this specialist herbivore, as larval mortality increases when the de-glucosylation is attenuated by silencing a specific larval beta-glucosidase by plant-mediated RNAi.^26^ Interestingly, preventing this de-glucosylation also increases larval unpalatability to native predatory spiders, suggesting that post-ingestive modifications can regulate sophisticated tritrophic interactions.^26^ Whether this de-glucosylation process of HGL-DTGs is effective against generalist herbivores remain an open question.

In addition to hydroxylation and de-glycosylation, another post-ingestive modification of HGL-DTGs is de-malonylation. In response to attack by herbivores, the malonylation of HGL-DTGs in plants is greatly enhanced by jasmonate signaling.^28^ However, the ester bonds that link the malonyl moiety and glucose is also readily cleaved under the high alkaline condition (pH > 8.5) of the *M. sexta* larval midgut, which suggests that the malonylation process likely does not factor into the defensive function of HGL-DTGs against caterpillars.^26^ Genetic manipulations of the malonyltransferase that controls this malonylation step revealed that the jasmonate enhanced malonylation maintains homeostasis of total malonylation ratios in response to the strongly induced biosynthesis of HGL-DTGs by jasmonate signaling^25^ and that unbalanced malonylation status affects stylar development and leads to plant sterility,^25^ highlighting a primary, physiological role for these specialized metabolites.

### 1.2 Post-ingestive metabolism by insect herbivores can reveal the function of PSMs

**Fig. 2:**
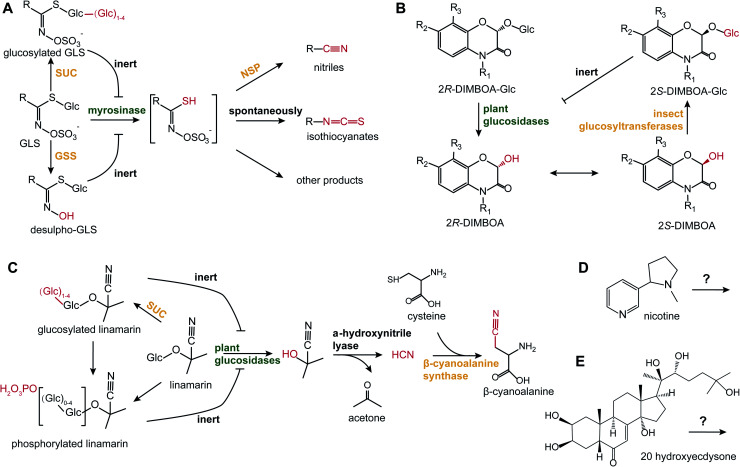
Post-ingestive modifications of defensive PSMs. (A) When plant cells are damaged, myrosinases cleave the glucose from GLS, generating the unstable GLS aglycone. Using nitrile-specifier proteins (NSP), insect herbivores can transform the aglycone to nitriles, which prevents their spontaneous transformations into more toxic isothiocyanates.^32^ Some other insects can directly modify GLS by adding extra glucoses *via* sucrase (SUC),^34^ or by de-sulphatation *via* glucosinolate sulphatase (GSS).^33^ (B) 2*R*-DIMBOA-Glc is converted into toxic aglycone, 2*R*-DIMBOA, by plant glucosidase. The 2*S*-DIMBOA, epimer of 2*R*-DIMBOA, can be re-glucosylated by insect glycosyltransferases to produce the less toxic epimer, 2*S*-DIMBOA-Glc that is inert to plant glucosidase.^36^ (C) Linamarin, a representative cyanogenic glucoside, is hydrolyzed by plant glucosidases and hydroxynitrile lyase to liberate HCN. Insects can transform HCN into β-cyanoalanine by conjugating cysteine *via* β-cyanoalanine synthase.^38^ Whiteflies can modify linamarin with extra glucoses and/or phosphates to prevent the formation of hydrogen cyanide.^39^ (D and E) Illustrate PSMs with specialized targets: (D) nicotine and (E) 20 hydroxyecdysone. Text in green and orange indicates enzymes from plants and insects, respectively. Red highlights moieties that are subjected to transformation. Inhibition symbols indicate the chemicals are inert to the corresponding enzymes.

In addition to HGL-DTGs, post-ingestive modifications have been reported for all the major examples of two-component defenses, such as glucosinolates (GLS), benzoxazinoids, cyanogenic glucosides and phenolics^23^ (Fig. 2). As they are the dominant defensive compounds in model plant, *Arabidopsis*, both GLS biosynthesis and metabolism have been intensively studied. GLSs and their detonator, myrosinases, are stored in separate cells, S-cells and myrosin idioblasts, which upon damage from both chewing and piercing-sucking insects, leads to the cleavage of the GLS glucose moiety.^30^ The resulting unstable aglycone is converted to a variety of metabolites by a variety of mediated and spontaneous rearrangements: spontaneously to form isothiocyanates (ITC); to nitriles by nitrile-specifier proteins from insects; to epithionitriles by epithio-specifier proteins in plants.^31^ Nitrile-specifier proteins that mediate nitrile formation can split the unstable aglycone from ITC to nitrile as a detoxification product, as nitrile is less toxic than ITC to insects.^32^ By removing the sulphate moiety from GLS by a sulphatase, crucifer specialist insects can disarm the “mustard oil bomb” by preventing the de-glucosylation and thereby, activation, by myrosinases.^33^ Frass metabolite analyses of the frass from whitefly piercing-sucking insects, called “honeydew”, revealed that phloem-feeding insects can also activate the “mustard oil bomb” and detoxify GLSs through the addition of an extra glucose at the GLS glucose moiety which also prevents de-glycosylation by plant myrosinases.^34^

Benzoxazinoids are another class of glycoside two-component PSMs that are the most important class of defensive compounds against herbivores of cereal crops. Through unbiased metabolomics screening of pre- and post-digested plant tissues, de-glucosylated benzoxazinoid aglycone and re-glucosylated products were detected from damaged plant tissues and herbivore frass, respectively, representing the activation and detoxification of these two-component defenses.^35^ Notably, the re-glucosylated benzoxazinoid, (2*S*)-2-β-d-glucopyranosyloxy-4-hydroxy-7-methoxy-2*H*-1,4-benzoxazin-3(4*H*)-one [(2*S*)-DIMBOA-Glc], in the frass of *Spodoptera* larvae is an epimer of ingested plant (2*R*)-DIMBOA-Glc and inert to plant glucosidase.^36^ Interestingly, the root-excreted benzoxazinoids can form complexes with iron in soil that facilitate iron uptake, which is further exploited by root-feeding insect herbivores and used to localize their host plants.^37^ For cyanogenic glucosides, the highly toxic hydrogen cyanide is released by beta-glucosidases when tissues are damaged. This hydrogen cyanide can further be converted with cysteine into cyanoalanine by beta-cyanoalanine synthase, representing a detoxification procedure in cyanide-resistant specialist insects, such as the burnt moth.^38^ Additionally, the whitefly *Bemisia tabaci* can conjugate glucoses and/or phosphates onto linamarin, a dominate cyanogenic glucosides in cassava, to prevent the generation of hydrogen cyanide from ingested linamarin.^39^

In addition to these two-component compounds, many defensive compounds are further modified after insect ingestion. Phenolics can also be further modified, such as by malonylations in the guts of polyphagous whitefly.^40^ Interestingly, the gene coding for the enzyme catalyzing the malonylation plausibly originates from plants through horizontal gene transfer.^40^ Triterpenoids can be further metabolized by root microbes when excreted into soil;^41^ sesquiterpene lactones can be modified in the gut of root herbivore *Melolontha meloontha* by a β-glucosidase that mediates the deterrence of PSMs;^42^ trichome-derived acylsugars are cleaved into sugar and volatile branch-chain fatty acids in the guts of *M. sexta*, and when these volatile aliphatic acids are released from frass, they attract predators, such as the omnivorous ant *Pogonomyrmex rugosus*^43^ as well as ground-feeding lizards.^44^ All these examples are consistent with the inference that plant-accumulated PSMs are frequently not the final functional products, that the post-ingestive modifications of PSMs, especially for those two-component compounds of generalized targets, are essential for the ecological functions of PSMs. Whether PSMs of specialized targets, such as nicotine and phytoecdysteroids, also undergo post-ingestive modifications to enhance toxicity or be counteracted during plant-insect interactions, is less understood and needs additional research to clarify (Fig. 2).

### 1.3 Post-ingestive metabolite interactions

Metabolite mixtures affect multiple pharmacological targets and provide clinical efficacy frequently superior to that of single compound-based drugs. Mixtures can also decrease the incidence of resistance,^45^ and provide compound synergisms. In ecological interactions, metabolite combinations can benefit defensive efficacy and be an important evolutionary force increasing metabolic diversity in plants.^14,15,46^ These interactions can happen among members of the same PSM class, as has been shown for acylsugars,^47^ or among metabolites from distinct biosynthetic pathways, such as between nicotine and proteinase inhibitors.^13^

However, the increased structural diversity in PSMs may not always be beneficial for the producers, as recently revealed by the discovery of a class of novel compounds that contain moieties of HGL-DTGs and hydroxycinnamoyl in the frass of *M. sexta* larvae that fed on *N. attenuata* plants.^29^ Bioassays with chemical-supplemented artificial diets and transgenic plants, in which the biosynthesis of specific metabolites were attenuated, revealed the origins of these novel metabolites: the hydroxycinnamoyl moieties, specifically caffeoyl, coumaroyl and feruloyl, were from hydroxycinnamoyl quinate conjugates, chlorogenic acids, a class of universally distributed phenolics. Functional analysis revealed that the interaction between HGL-DTGs and phenolics impedes the defensive function of both PSM classes, and is a detoxification strategy of the specialist *M. sexta*. Chlorogenic acid has long been known as a dual-function metabolites, providing protection from both herbivores and UV-B irradiation.^48,49^ Probably as a consequence of the long history of arms races with specialist herbivores, the other part of chlorogenic acid, quinate, can also be esterified with the salicylic acid moiety of salicortin, a dominate defensive phenolic compound in Salicaceae plants, after digested by a specialist lepidopteran herbivore *Cerura vinula*, again, likely representing a detoxification process.^50^ These examples of post-ingestive metabolism between two PSM classes reveal that metabolic diversity can be co-opted by specialist herbivores, and provides a cautionary tale for the possible drawbacks of metabolite diversity when mixtures are used in medicines and insecticides. Notably, this cautionary tale is also consistent with the results of a recent meta-analysis that revealed that synergistic effects among PSMs are more often effective against generalist insect herbivores;^51^ specialist insects appear to evolve sophisticated detoxification mechanisms and can even co-opt the defensive value of PSM diversity.

### 1.4 Future directions for functional studies of PSMs

Post-ingestive modifications are important steps for insect detoxifications of PSMs and may contribute to the surge of insecticide resistance in agriculture, similar to the discovery of the natural origins of the antibiotic resistance in the virulent pathogens that now inhabit hospitals and other facilities where antibiotics are commonly used. For example, the penicillin resistance gene penicillinase, a β-lactamase that inactivates penicillin, was identified before the introduction of penicillin as a therapeutic.^52^ Whether the exuberant use of antibiotics in medicine and agriculture is responsible for the origins of antibiotic resistance is still disputed,^53^ but examples of both antibiotic and insecticide resistances originating from standing variation in natural populations are being reported.^54,55^ Aphids are polyphagous insects that speciated into different races based on different host plants. A race (*Myzus persicae nicotianae*) that adapted to feed on tobacco easily evolved resistance to neonicotinoid pesticides.^56^ A recent meta-analysis revealed that most examples of herbicide resistance and a large portion of insecticide resistances evolve through selection of pre-existing genetic variation.^57^ From these emerging trends, we infer that a deeper appreciation of post-ingestive modifications of PSMs is likely to provide valuable insights and predictions about the emergence of insecticide resistance in agricultural pests.

**Fig. 3:**
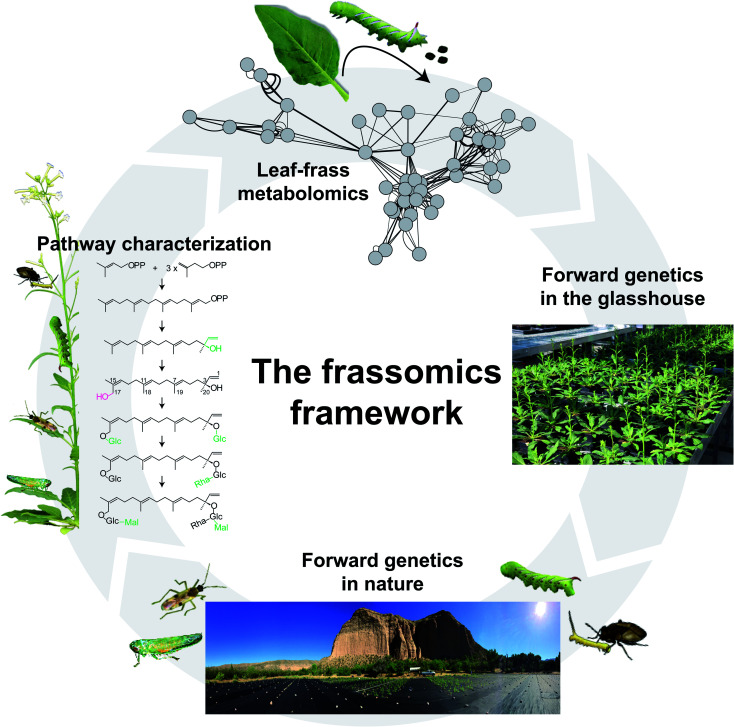
An insect-guided forward-genetics framework to study the function of PSMs. As described in Fig. 1, comparative leaf and frass metabolomics can elucidate the post-ingestive modification of PSMs, and contribute to our understanding of PSM functions in both producers and consumers of PSMs. In addition to the reverse-genetics approaches used in a frassomics context (for examples, see references in Fig. 1 caption), forward-genetics tools, such as the Recombinant Inbred Lines (RILs) that can capture a majority of the natural variation in a species in a structured manner (*e.g.*, Multiparent Advanced Generation Intercross (MAGIC) RIL populations) can be used in a frassomic context to conduct frass metabolite QTL (fmQTL) imputations that can bridge the gap between frass metabolites and plant genetics, and provide a powerful tool with which to identify plant enzymes that control post-ingestive modification of PSMs. When released into natural habitats, these forward genetic tools can be used to study the frassomics of multitrophic interactions mediated by PSMs.^64^

The rapid advances in high-resolution mass spectrometry have accelerated the discovery of *m*/*z* features indicative of post-ingestive novel compounds through comparative metabolomics analysis of frass and plant samples. However, similar to the research in plant metabolomics, elucidating chemical structures of the vast unknown post-ingestive compounds, which is important for functional studies, remains a major challenge. The frassomics analysis^21^ that uses structural similarity clustering of all spectra, detected associations of unknown post-ingestive metabolites with known plant metabolites, thereby illuminating the metabolic origins of many novel frass metabolites likely involved in the plant defense/insect detoxification processes (Fig. 3). Mutant plants that lack the ability to synthesize known PSMs are invaluable tools to impute the origins of post-ingestive novel compounds derived from the manipulated pathways using comparative frassomics of herbivores feeding on mutant and wild type plants. However, such comparative analyses need to be grounded by prior knowledge of metabolite biosynthesis in plants, which is largely lacking in the PSM-replete non-model plants.

Post-ingestive modifications frequently result in fluctuations of transcripts and proteins catalyzing the ingested metabolites in coordination with the ingested quantities. Thus, comparative transcriptomics of herbivore guts containing different quantities of PSMs (by feeding on naturally varying plants or engineered mutants), can provide valuable information about genomic loci that could be targeted for manipulations by gene editing or plant mediated RNAi.^10^ This approach is the classical reverse-genetic approach, which is relatively straightforward but limited in its ability to identify novel genes and pathways. In contrast, forward-genetics is a promising approach for the elucidation of unknown genes.

Forward-genetic approaches, such as bulked segregant analysis,^58^ using herbivore populations that harbor different modification abilities, can link the quantitative variation in post-ingestive metabolite accumulations with genetic variation in herbivores. When post-ingestive modifications are mediated by plant enzymes, as discussed for the hydroxylation processes that render HGL-DTGs toxic in the guts of *M. sexta* larvae, normal forward-genetic approaches using genetically structured herbivore populations will be of little use. Quantitative trait loci (QTL) mapping is another widely used forward genetic tool in plant science to impute genetic loci using recombinant inbred lines (RILs) to capture a majority of the natural variation in a species in a structured manner. For example, Multiparent Advanced Generation Intercross (MAGIC) RIL populations that are derived from intercrossing multiple parental accessions provide a powerful means of using genetic variances to bridge the gap between plant metabolites and plant genetics. However, the traditional metabolite QTL (mQTL) approach will not work when the genetics controlling metabolite modifications are regulated post-ingestively. Here, we propose a new concept, frass metabolite QTL (fmQTL) analysis which addresses this issue (Fig. 3). In this procedure, genetically homogenous herbivores fed genetically varied plants, will produce quantitative frass traits in which the variable intensities of metabolites modified in the frass or the ratio of metabolic modification can be mapped to the genome of the genetically varied plants. The fmQTL analysis can not only elucidate plant genetic loci that control the production of new metabolites in frass, but also be applied to metabolites that are not readily quantified in specific plant tissues. For example, the fmQTL approach can take advantage of the selective feeding behaviors of particular insect feeding guilds, such as phloem sap and mesophyll feeders, by focusing on the frass of phloem-sucking insects and leaf miners.

When the structures and the genes responsible for the post-ingestive modifications of PSMs are elucidated, the next steps could be the elucidation of the mechanisms of how defensive PSMs function, which frequently translates into understanding the PSM-targeted proteins. Most of current defensive functions of PSMs are defined by insect growth or performance bioassays, assays that provide few insights into the exact targets of PSMs. While transcriptome and proteome profiling after PSM ingestion can be very powerful, the patterns are commonly challenging to interpret given the regulatory chaos imparted to a biological system from toxin ingestion. Here the complete toolbox of chemical proteomics approaches developed in last decades to identify the proteins that directly interact with drug ligands, will be transformative.^59^ The general strategy of this approach is to link candidate compounds with a tag, such as biotin and alkyner, so as to fish target proteins, followed by protein identification by mass spectrometry.^60^ Based on the principle that protein melting curve profiles can be altered by interactions with small molecules, thermal proteome profiling represents another promising unbiased approach to screen cellular proteomes that interact with PSMs of interest using multiplexed quantitative mass spectrometry-based proteomics.^61^

A commonly used bioassay method is to test the defensive function of PSMs and their post-ingestive modifications by supplementing artificial diets. The advantages of artificial diets are that they offer defined dietary backgrounds. However, their disadvantages are becoming increasingly apparent. In some cases, as in the hydroxylations of HGL-DTGs that require an unknown plant constituent for toxin activation, the standard artificial diets for *M. sexta* larvae based on milk protein and wheat germ, are of little value, providing misleading information.^21^ Disinfectants, such as formalin, and antibiotics are essential components that enhance the shelf life of artificial diets, but these can severely confound functional interpretations, particularly when symbionts are involved.^62^ Most artificial diets contain unnaturally high protein and sugar levels to initiate larval acceptance, but these are known to modify PSM processing.^63^ Thus, performance bioassays with plants, genetically modified in their metabolite accumulations, provide a superior means of evaluating the defensive function of ingested PSMs.

Compared with the PSM biosynthesis in plants, few studies of PSMs function have been conducted in natural habitats, which means that PSM functions that engage interactions with other organisms are largely ignored. Considering that the raison d'etre^5^ of PSMs are thought to help plants adapt diverse environmental stresses, this is a serious issue, and a major motivation for the “natural history motivated gene function” research program, described above. This natural history guided approach can be an enormous boon for forward-genetics guided approaches, as exemplified by the recent discovery of new defense chemistry using this approach.^64^*N. attenuata* plants in nature interact with more than 30 different herbivore and pathogen taxa, and these can be used to screen large forward-genetic plant populations. Five of these interacting insects are being used as high-throughput screeners to phenotype traits of *N. attenuata* plants.^64,65^ Through their centuries of interactions with their host plants, these natural insect screeners have evolved uncanny abilities to discriminate amongst the genetics, metabolites and traits that can benefit their survival. The associations between metabolites and genetics can also contribute to the elucidation of biosynthesis and regulatory pathways (Fig. 3).

## 2 Author contributions

Jiancai Li, Ian T. Baldwin and Dapeng Li designed the structure of the review; Jiancai Li prepared the first draft which was revised by all authors.

## 3 Conflicts of interest

All authors have declared no competing interests.

## Supplementary Material
